# A longitudinal study of quality of life of earthquake survivors in L’Aquila, Italy

**DOI:** 10.1186/1471-2458-13-1143

**Published:** 2013-12-07

**Authors:** Marco Valenti, Francesco Masedu, Monica Mazza, Sergio Tiberti, Chiara Di Giovanni, Anna Calvarese, Roberta Pirro, Vittorio Sconci

**Affiliations:** 1 Department of Applied Clinical Sciences and Biotechnology, Section of Clinical Epidemiology and Environmental Medicine, University of L’Aquila, Via Vetoio, Coppito 2, 67100 L’Aquila, Italy; 2 Department of Life, Health and Environmental Sciences, University of L’Aquila, Piazza S. Tommasi, Coppito, 67100, L’Aquila, Italy; 3 Department of Mental Health, Territorial Health Agency ASL1, P.O. Collemaggio, 67100 L’Aquila, Italy

**Keywords:** Quality of life, Mental health, Earthquake, Disaster relief, Longitudinal studies, Psychosocial factors

## Abstract

**Background:**

People’s well-being after loss resulting from an earthquake is a concern in countries prone to natural disasters. Most studies on post-earthquake subjective quality of life (QOL) have focused on the effects of psychological impairment and post-traumatic stress disorder (PTSD) on the psychological dimension of QOL. However, there is a need for studies focusing on QOL in populations not affected by PTSD or psychological impairment. The aim of this study was to estimate QOL changes over an 18-month period in an adult population sample after the L’Aquila 2009 earthquake.

**Methods:**

The study was designed as a longitudinal survey with four repeated measurements performed at six monthly intervals. The setting was the general population of an urban environment after a disruptive earthquake. Participants included 397 healthy adult subjects. Exclusion criteria were comorbidities such as physical, psychological, psychiatric or neurodegenerative diseases at the beginning of the study. The primary outcome measure was QOL, as assessed by the WHOQOL-BREF instrument. A generalised estimating equation model was run for each WHOQOL-BREF domain.

**Results:**

Overall, QOL scores were observed to be significantly higher 18 months after the earthquake in all WHOQOL-BREF domains. The model detected an average increase in the physical QOL scores (from 66.6 ± 5.2 to 69.3 ± 4.7), indicating a better overall physical QOL for men. Psychological domain scores (from 64.9 ± 5.1 to 71.5 ± 6.5) were observed to be worse in men than in women. Levels at the WHOQOL domain for psychological health increased from the second assessment onwards in women, indicating higher resiliency. Men averaged higher scores than women in terms of social relationships and the environmental domain. Regarding the physical, psychological and social domains of QOL, scores in the elderly group (age > 60) were observed to be similar to each other regardless of the significant covariates used.

**Conclusions:**

WHOQOL-BREF scores of the psychological domain displayed trends conditioned by age and education: older subjects experienced less satisfaction with psychological health on average. Less-educated subjects always demonstrated the worst QOL scores. Gender, age and education impacted the variability of QOL in the environmental dimension in the elderly.

## Background

On 6 April 2009, a catastrophic earthquake seriously damaged the city of L’Aquila in the Abruzzo region of Italy. The population faced loss of homes, with 67,000 persons displaced to the region’s coast or forced to live in tents. The destruction wrought by the earthquake resulted in 308 deaths and numerous injuries. The social and psychological impact on the entire community was immense [1,2].

Earthquakes occur without warning and give the population no opportunity to make psychological adjustments to face the calamity [3]. The lack of predictability, the reminders of the destruction and the need to move due to the destruction of homes may result in effects ranging from discouragement to serious mental health issues by exacerbating the emotional reactions associated with the trauma [4].

Many studies on the psychological health of seismic victims have assessed the long-lasting consequences of earthquakes on psychological health based on age, gender, education and social conditions [5-8].

Many studies have emphasised that earthquakes impair the subjective quality of life (QOL) of survivors [9,10], and several longitudinal studies have been conducted to identify determinants of poor QOL [8]. The association between poor QOL and earthquake-induced post-traumatic stress disorder (PTSD) has been widely investigated in recent literature [8,11]. People’s well-being after loss resulting from an earthquake is currently a concern in countries exposed to natural disasters both for public health reasons and for the economical sustainability of specific interventions [12,13].

However, most studies on post-earthquake QOL have focused on the effects of psychological impairment and PTSD on the psychological dimension of QOL. However, to better understand the dynamics of a population’s health in the aftermath of a disaster, studies must focus on QOL in populations not affected by PTSD or psychological impairment [14].

The goal of the current study was to assess and estimate the QOL changes that occurred in an adult disease-free population sample after the 2009 earthquake in L’Aquila, Italy and to examine the relationship between changes in QOL and gender, age, socio-economic status and education by using a population-averaged statistical model.

## Methods

### Design and participants

For 18 months after the earthquake, we monitored the QOL of a sample of 397 subjects (210 females, 52.9%; and 187 males, 47.1%) older than 18 years and living in L’Aquila at the time of the earthquake. Participants represented nearly 80% of the 500 subjects (i.e., approximately 1% of the adult population of L’Aquila) selected for the study based on multi-stage random sampling. The 19 new towns in the outskirts of L’Aquila represented the first sampling stage and their populations the second sampling stage. Among the original 500 subjects, 84 were excluded because of the presence of comorbidities such as physical, psychological, psychiatric or neurodegenerative diseases at the beginning of the study, and 19 refused to participate.

The study was designed as a longitudinal survey with repeated measurements of QOL at four times: November 2009, May 2010, November 2010 and May 2011.

The following set of socio-demographic explanatory variables was recorded at the time of the first interview: gender, age (<40; 40–60; >60 years), education (primary school, secondary school, degree), marital status (single, married, separated/widower), parenthood and employment status (employed, unemployed, housewife, student). Socio-economic status was characterised using dwelling size in square metres as a continuous variable.

The questionnaires were consistently administered individually by the same team of four professional psychologists, each of whom received specific training on the instrument. Adequate matching of the subjects was ensured by the experimenters. Twenty seven out of 397 participants (6.8%) dropped out during the second administration of the questionnaire, 31 out of 370 (8.4%) dropped out during the third administration and 34 out of 339 (10%) dropped out during the last administration. Dropouts were due both to lack of participant compliance to remain in the study and to the occurrence of the exclusion criteria defined above.

A written informed consent form was signed by all participants. The study was approved by the advisory board of the Department of Mental Health of L’Aquila Health Agency and conducted according to the Helsinki Declaration.

### Measurements

QOL was assessed by the Italian version of the World Health Organization Quality of Life BREF assessment instrument (WHOQOL-BREF) [15,16]. The WHOQOL assessment is a cross-culturally valid assessment of well-being. The WHOQOL-BREF was developed as a 26-item version of the WHOQOL-100 instrument for use in situations in which time is restricted and respondent burden must be minimised, such as in epidemiological surveys [17]. The WHOQOL-BREF is a person-centred instrument designed for use as a multi-dimensional profile, enabling a wide range of conditions to be compared. It is a generic instrument, encompassing health-related and contextual issues, plus a general facet on health and overall QOL. The instrument consists of QOL items that concern the meaning of different aspects of life to the respondents and how satisfactory or problematic their experience is. The WHOQOL-BREF provides an assessment of QOL in four domains, each structured in relevant items: physical health (seven items), psychological (six items), social relationships (three items), and environmental (eight items). Two further items concern a subjective scoring of overall QOL and health. Each item contributes to the calculation of the overall domain score, ranging from 0 to 100, to enable comparisons between domains composed of an unequal number of items [18].

### Data analysis

A preliminary graphical survey of the average trend associated with different QOL domains, depending on the configuration of different covariates, was performed, and a background for the plausibility of further analysis was set.

A generalised estimating equation model (GEE) was run for each domain, regressing the correspondent QOL scores on the explanatory variables chosen, so that the population average mean response could be assessed according to different profiles [19-21]. The choice of a GEE model instead of a repeated measures ANOVA does not require sphericity hypotheses about the covariance structure. In addition, GEE models address drop-out issues, incorporating all available pairs into the estimation of the working correlation parameters as well as those derived from records with missing data. An unstructured working correlation was selected to minimise the assumptions. The Wald test of statistical significance for regression coefficients, with a confidence level set to 5%, was performed [22].

The analysis was carried out using the statistical software STATA 11.

## Results

The mean age of the participants was 52.2 years (standard deviation 7.1 years). The sample included 88 subjects (22.2%: 34 women, 54 men) with primary education, 211 subjects (53.1%: 120 women, 91 men) with secondary education and 98 (24.7%: 56 women, 42 men) with degree-level education such that women on average had a higher education level than men, consistent with data from the general population survey administered in the same area. With respect to marital status, 116 (29.2%) participants were not married, 225 (56.7%) were married and 56 (14.1%) were separated or widowed. Two hundred twenty subjects (55.3%) were currently employed, and 177 (44.7%) were unemployed, housekeepers or students.

Table 1 reports QOL scores, by domain, across the four follow-up times with no stratification by covariates. Overall, the QOL scores were significantly higher 18 months after the earthquake in all WHOQOL-BREF domains.

**Table 1:** WHOQOL-BREF scores at four follow-up times

	**Follow-up (Mean ± SD)**				
**WHOQOL-BREF domains**	**November 2009**	**May 2010**	**November 2010**	**May 2011**	**Paired *t*-test p-value**
**Physical activity domain**	66.62 ± 5.23	66.75 ± 6.23	66.08 ± 5.27	69.36 ± 4.78	<0.05
**Psychological domain**	64.90 ± 5.18	65.64 ± 5.83	68.57 ± 6.29	71.49 ± 6.56	<0.01
**Social relationships domain**	70.17 ± 6.20	69.16 ± 5.58	69.96 ± 5.17	73.32 ± 4.10	<0.05
**Environmental domain**	63.67 ± 5.43	62.85 ± 5.40	66.17 ± 6.03	66.01 ± 4.80	<0.05

The mean scores along time for each dimension of the questionnaire are reported. Paired *t*-test compares baseline (November 2009) to final (May 2011) scores.

Table 2 reports the average covariate effect over time on the four WHOQOL-BREF domain scores. In the analysis, variables demonstrating collinearity were omitted to avoid possible inflation of the standard deviations: socio-economic status was collinear with education, parenthood with marital status and employment with education.

**Table 2:** GEE marginal expectation model of WHOQOL-BREF domains (cell values are beta regression coefficients ± standard error)

**Covariates**	**Physical activity domain**	**Psychological domain**	**Social relationships domain**	**Environmental domain**
**Gender**	0.89 ± 0.19	−1.16 ± 0.31	1.22 ± 0.20	0.98 ± 0.33
**Age**	11.79 ± 0.91	3.81 ± 1.01	−1.41 ± 1.09 ns	1.65 ± 1.25 ns
**Time**	−0.76 ± 0.54 ns	2.96 ± 0.52	2.93 ± 0.60	1.34 ± 0.62
**Education**	2.74 ± 0.96	5.48 ± 1.25	−3.49 ± 1.16 ns	−2.22 ± 1.30 ns
**Marital status**	0.12 ± 0.1 ns	0.07 ± 0.22 ns	−0.18 ± 0.11 ns	−0.28 ± 0.20 ns
Time * Age	0.32 ± 0.14	−0.95 ± 0.15	−0.28 ± 0.17	−0.11 ± 0.16 ns
Time * Education	0.54 ± 0.15	0.57 ± 0.17	−0.68 ± 0.19	−0.05 ± 0.18 ns
**GEE model constant**	57.55 ± 0.85	60.18 ± 1.12	70.68 ± 1.03	61.56 ± 1.16

All coefficient values are statistically significant unless otherwise specified (ns = not significant).

The covariate marital status was never statistically significant; thus, we could not consider it as a determinant of QOL in any of the dimensions studied. Another common feature of the results was the dependence on gender, which performed differently within the questionnaire dimensions. For instance, the model detected an average increase in the physical QOL score in men (Mean men = 68.18, SD men = 5.32; Mean women = 67.26, SD women = 5.63; B_Gender_ = 0.89) (Table 2), indicating better overall physical QOL of men after such a natural disaster, but men behaved worse than women with respect to the psychological QOL score (Mean men = 66.66, SD men = 6.06; Mean women = 68.48, SD women = 6.78; B_Psychological_ = −1.16). We observed, given the other conditions, a better psychological resiliency for women, who displayed reactions since May 2010, more than one year after the earthquake (Table 1).

Men averaged higher WHOQOL scores compared to women in terms of social relationships (Mean men = 71.25, SD men = 5.32; Mean women = 70.08, SD women = 5.69; B_social relationships_ = 1.22) and the environmental dimension (Mean men = 65.18, SD men = 5.32; Mean women = 64.20, SD women = 5.65; B_environmental_ = 0.97).

**Figure 1:**
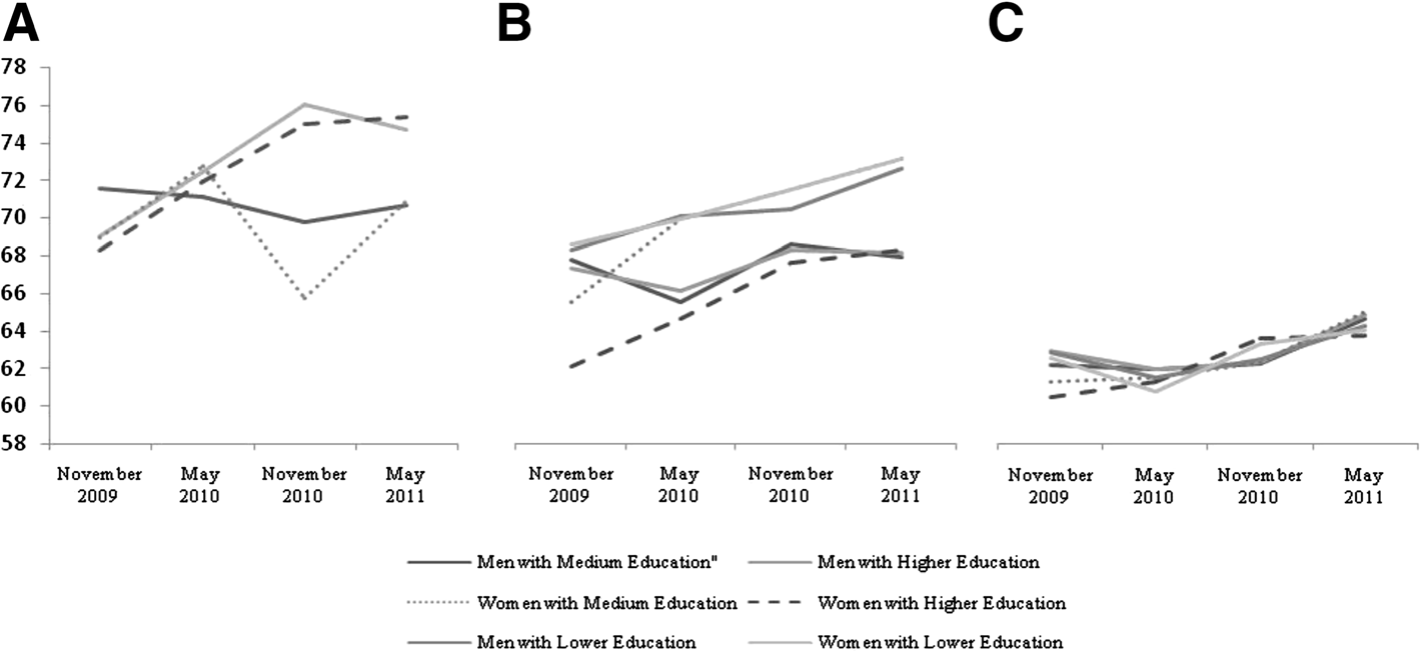
**WHOQOL-BREF Physical activity scores mean trends. (A)** Age < 40 **(B)** Age 40-60 **(C)** Age≥60.

**Figure 2:**
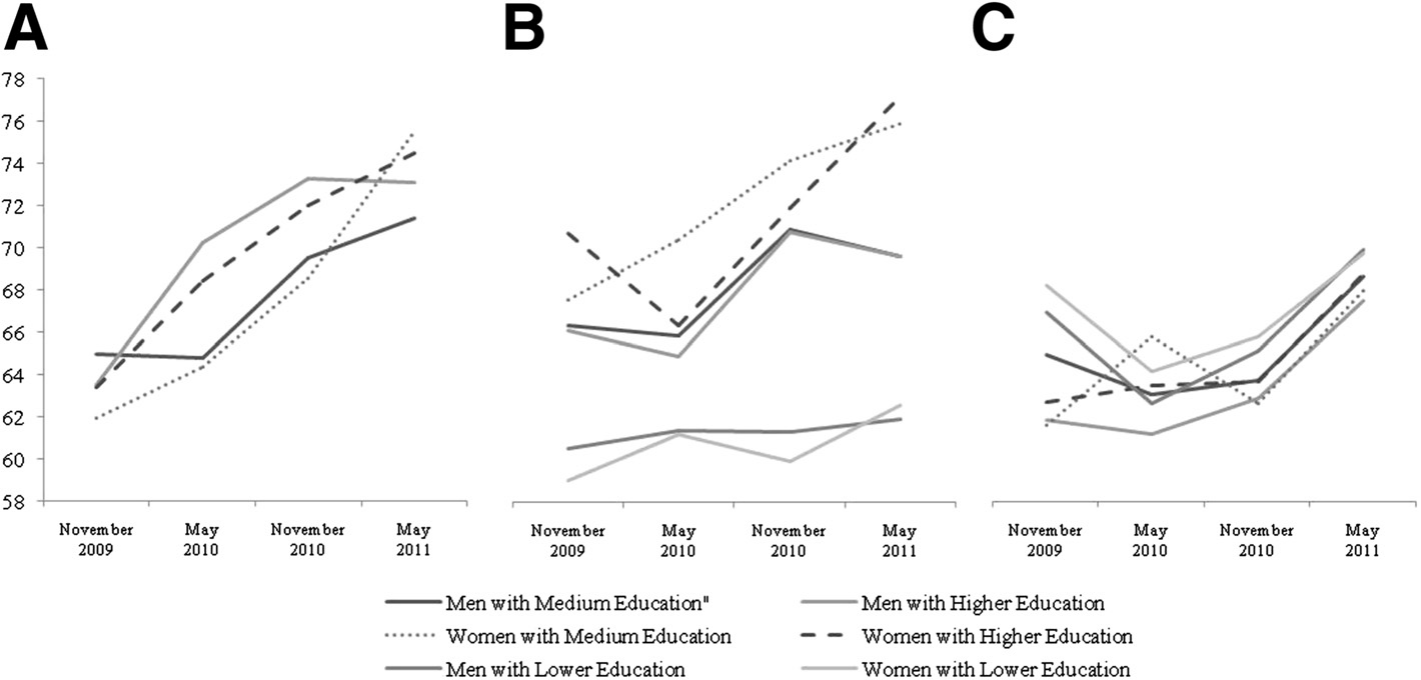
**WHOQOL-BREF Psychological activity scores mean trends. (A)** Age < 40 **(B)** Age 40-60 **(C)** Age≥60.

**Figure 3:**
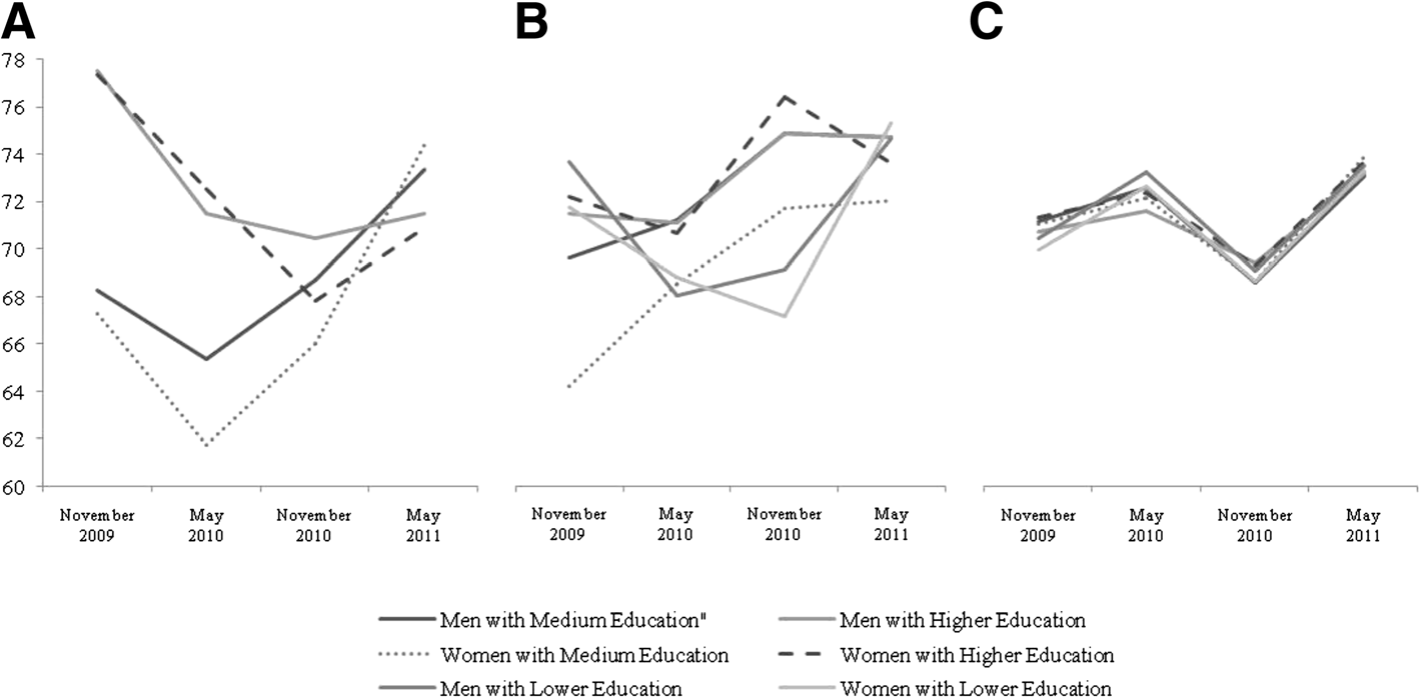
**WHOQOL-BREF Social relationships scores mean trends. (A)** Age < 40 **(B)** Age 40-60 **(C)** Age≥60.

The details of the GEE marginal expectation model, provided for each dimension of the questionnaire (Table 2), show different response patterns over time (Figures 1, 2 and 3). The analysis revealed a clear interaction between time and education (B _Time*Education_ = 0.54) and time and age (B _Time*Age_ = 0.32) in the physical activity domain. The interaction between time and age disappeared in the case of the social relationships area (p > 0.102). No time per gender interactions was statistically significant in any questionnaire area. The covariate time showed weight changes according to the questionnaire dimensions, demonstrating that the psychological area was on average the most affected by time (B_Time_ = 2.96).

## Discussion

After a natural disaster, QOL profiles and their determinants exhibit a manifold pattern strongly influenced by many factors [23].

Our study accounted for a wide set of possible factors representing specific areas of relative vulnerability and strength of the population. In the following, we discuss some of the resulting qualified patterns. As shown in Table 1, participants had mostly lower QOL at the different intervals (except the social relationships domain) compared with the QOL of European populations, where the mean scores for the subjective QOL of a healthy general population is >70% on a scale from 0 to 100 [24]. One possible explanation for this finding relies on the impact that a disaster such as the L’Aquila earthquake also has on healthy general populations.

The WHOQOL-BREF scores of the psychological domain displayed trends conditioned by age and education such that older subjects exhibited a greater variation in post-trauma QOL scores (Figure 2c). In contrast, subjects less than 40 years old showed an overall increasing monotonic trend, with some variations according to gender and education level (Figure 2a). Regarding people between 40 and 60 years of age, education level produced time-dependent responses, with better QOL scores observed for those with medium and higher education. Less-educated subjects, irrespective of gender, showed time-insensitive responses, with a nearly flat trend. It is worth noting that, despite this apparent advantage, these subjects always demonstrated the worst QOL scores to a great extent (Figure 2b).

The social relationships score displayed the opposite tendency of that observed for the previous questionnaire domains in terms of time dependence and variability. Increased response variability in the younger and middle age strata emerged, as corroborated by graphical evidence and GEE outcomes, showing a decreasing variability trend across strata (Figure 3).

**Figure 4:**
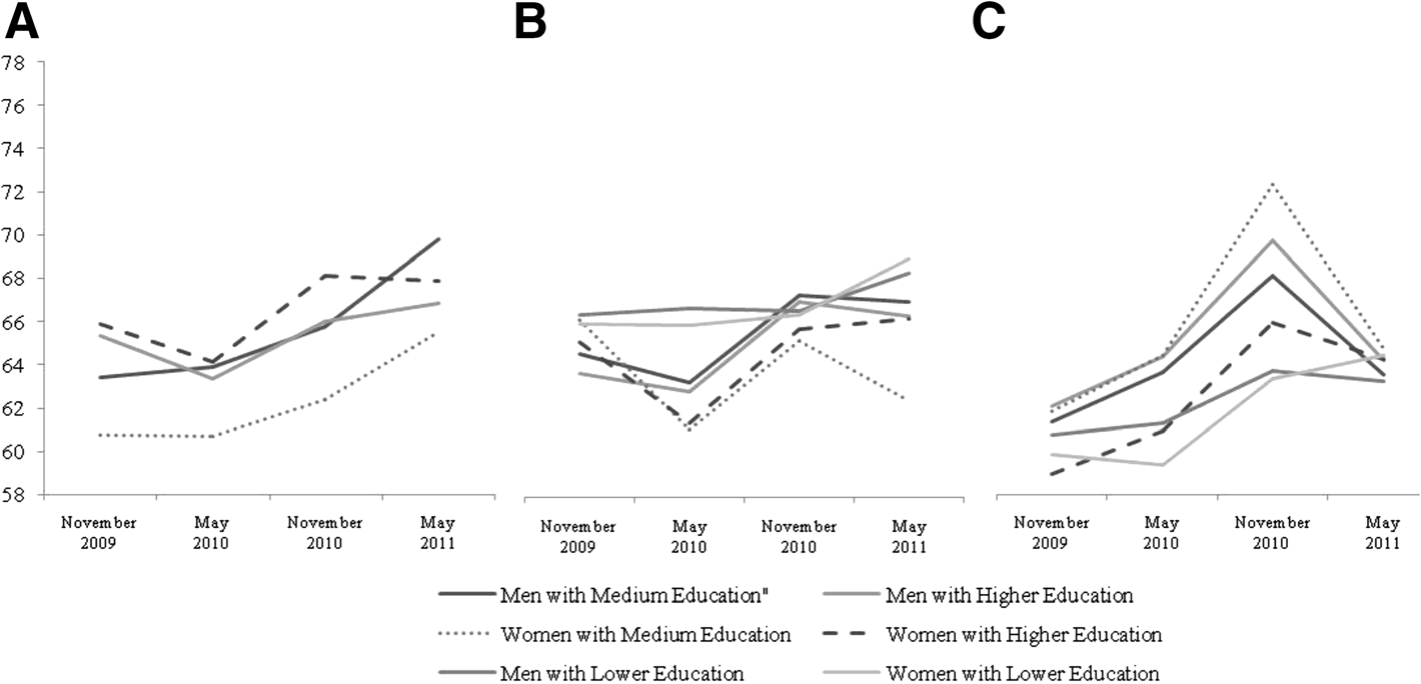
**WHOQOL-BREF Environmental scores mean trends. (A)** Age < 40 **(B)** Age 40-60 **(C)** Age≥60.

It is worth emphasising the low variability of QOL scores in the elderly across the physical, psychological and social dimensions of QOL (Figures 1c, 2c and 3c) compared to the other age groups. In contrast, the results demonstrated that covariates impact the variability of QOL in the environmental dimension (Figure 4c).

This study attempted to describe the profile trends of subjective QOL in a population sample exposed to a disruptive earthquake but not showing symptoms of psychological impairment. To date, the literature has widely described the strong association between PTSD or other psychological impairment and QOL but has not investigated QOL profiles in exposed healthy subjects after an earthquake. This line of research is particularly important for the management of public health interventions, especially with respect to the need for optimal resource allocation. Our multidimensional analysis can offer guidelines regarding what social groups are at a high risk of declining QOL, revealing areas of vulnerability identified by covariates in the aftermath of a disruptive natural disaster.

Several limitations affect this study. First, we lack data from before the earthquake; thus, it cannot be assumed that QOL outcomes were determined directly by the earthquake or were influenced by pre-existing time trends. Moreover, we could not analyse data concerning any changes in socio-demographic indexes (marital and employment status) following the earthquake. Second, the study could not benefit from a comparison with the QOL of non-disaster areas. Third, we could not consider the role played by social support, which has been described as a moderator between psychological discomfort and QOL [11].

## Conclusions

Despite its limitations, this study is sufficiently powerful to confirm the results of reports on QOL regarding the necessity of a complex frame of reference after a disaster, in which political decision-makers and public health institutions should consider population QOL as a main issue [25]. Moreover, we recommend further empirical studies with a larger sample size and prospective design to unravel the complexities of the QOL analysis as well as adequate public health preparedness as a central strategy in post-disaster management [26,27].

## Competing interest

The authors declare that they have no competing interests.

## Authors’ contributions

MV conceived and designed the epidemiological study, participated in its coordination and helped to interpret the statistical analysis results and draft the manuscript. FM performed the statistical data analysis and contributed to the drafting of the manuscript. MM contributed to data analysis and interpretation of psychometric dimensions of the instruments and contributed to the drafting of the manuscript. ST and VS participated in the design, conduct and coordination of the study and contributed substantially to data interpretation and the drafting of the manuscript. CDG, AC and RP contributed to instrument administration and the assignment of WHOQOL instrument scores and contributed to the drafting of the manuscript. All authors read and approved the final manuscript.

## Pre-publication history

The pre-publication history for this paper can be accessed here:

http://www.biomedcentral.com/1471-2458/13/1143/prepub
